# The Re-Emergence of H1N1 Influenza Virus in 1977: A Cautionary Tale for Estimating Divergence Times Using Biologically Unrealistic Sampling Dates

**DOI:** 10.1371/journal.pone.0011184

**Published:** 2010-06-17

**Authors:** Joel O. Wertheim

**Affiliations:** Department of Pathology, University of California San Diego, San Diego, California, United States of America; BC Centre for Excellence in HIV/AIDS, Canada

## Abstract

In 1977, H1N1 influenza A virus reappeared after a 20-year absence. Genetic analysis indicated that this strain was missing decades of nucleotide sequence evolution, suggesting an accidental release of a frozen laboratory strain into the general population. Recently, this strain and its descendants were included in an analysis attempting to date the origin of pandemic influenza virus without accounting for the missing decades of evolution. Here, we investigated the effect of using viral isolates with biologically unrealistic sampling dates on estimates of divergence dates. Not accounting for missing sequence evolution produced biased results and increased the variance of date estimates of the most recent common ancestor of the re-emergent lineages and across the entire phylogeny. Reanalysis of the H1N1 sequences excluding isolates with unrealistic sampling dates indicates that the 1977 re-emergent lineage was circulating for approximately one year before detection, making it difficult to determine the geographic source of reintroduction. We suggest that a new method is needed to account for viral isolates with unrealistic sampling dates.

## Introduction

Phylogenetic inference is an important tool for understanding the origin and evolution of emerging pathogens [1]. For rapidly evolving pathogens, such as RNA viruses, isolates sampled over years or decades can be used to calibrate a molecular clock and date divergence events [2]. However, if frozen laboratory strains escape into the general population, they will be missing years of nucleotide sequence evolution, and the date of isolation can be misleading.

**Figure 1:**
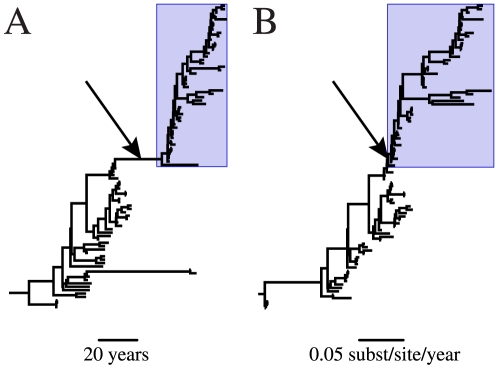
Maximum clade credibility phylogeny of human H1N1 influenza virus HA segment with unadjusted sampling dates. The topologies of (A) a chronogram in which branch lengths represent time and (B) a phylogram in which branch lengths represent nucleotide substitutions are identical. Avian and swine influenza virus lineages were removed for ease of viewing. Arrows indicate the lineage leading to the re-emergent HIN1 clade; boxes designate the re-emergent H1N1 clade.

The most famous case of a released laboratory strain is the re-emergent H1N1 influenza A virus which was first observed in China in May of 1977 and in Russia shortly thereafter [3], [4]. This outbreak marked the return of a seasonal H1N1 human influenza virus after a nearly 20-year absence following its displacement during the 1957 H2N2 pandemic. Scientists quickly realized that something was unusual about this re-emergent H1N1 strain; it was genetically similar, though not identical, to an H1N1 isolate from 1950 [5], [6]. Initially it was suggested that this virus could have lain dormant or evolved slowly in non-human hosts for decades, but it is now generally assumed that the virus was kept frozen in a yet unidentified laboratory [7], [8]. The glaring discrepancy between the amount of inferred evolutionary time (Figure 1A) and amount of sequence evolution (Figure 1B) leading to the 1977 outbreak provides evidence supporting this conclusion.

In a recent paper estimating the age of human pandemic influenza, Smith et al. [9] included the re-emergent H1N1 sequences in their analysis without correcting for the missing years of evolution. Here, we investigated the effect of including sequences with biologically unrealistic sampling dates on the ability to estimate the time of most recent common ancestor (tMRCA) in influenza virus.

## Analysis and Discussion

First, the amount of evolutionary time missing from the branch leading to the re-emergent H1N1 clade was inferred by examining the discrepancy between the root-to-tip genetic distance and sampling-year in the re-emergent H1N1 clade. This method was possible because influenza virus experiences a steady rate of sequence evolution [10]. Maximum likelihood phylogenies were constructed for each influenza virus genome segment in PHYML [11] under a GTR+Γ_4_ substitution model using sequences and rooting from Smith et al. [9]. For each segment, we calculated the distances between the regression line intercepts for the root-to-tip genetic distance versus sampling year for the re-emergent H1N1 lineage and the other human influenza viruses (pre-1977 H1N1, H2N2, and H3N2) (for method see [12]). Isolates in which sampling year was not consistent with the amount of sequence evolution (identified in [13], [14]) were removed from these analyses.

**Figure 2:**
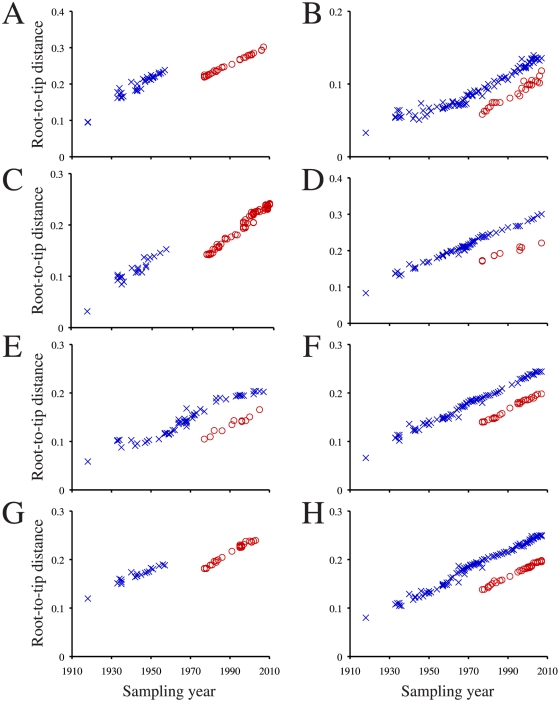
Root-to-tip genetic distance versus sampling year for human influenza virus segments. (A) HA, (B) M, (C), NA, (D) NP, (E), NS, (F) PA, (G) PB1, and (H) PB2 segments are shown. Pre-1977 H1N1, H2N2, H3N2 isolates are indicated with blue Xs, and re-emergent H1N1 isolates are indicated with red Os.

There was a clear shift in the root-to-tip distance in the re-emergent H1N1 clade. The slope of the re-emergent lineage and the other human influenza virus had mean/median difference of 27 years (Figure 2), suggesting that the virus was frozen for approximately 27 years before it re-emerged (e.g., virus isolated in 2007 is missing 27 years of mutations).

To determine how the “missing” evolution affected dating estimates, Bayesian Markov chain Monte Carlo (BMCMC) phylogenetic inference was performed on each genome segment for human influenza viral sequences (not including the 2009 H1N1 pandemic) and related non-human viral sequences in BEAST v1.5.2 [15]. The first analysis used the same sequences and sampling dates as Smith et al. [9]. The second analysis used the same sequences but adjusted the age of all H1N1 viruses isolated in or after 1977 by shifting their sampling date 27 years earlier. Multiple independent BMCMC analyses were run for each genome segment; convergence and adequate mixing (effective sample size of all relevant parameters >200) was verified in Tracer v1.5 (http://tree.bio.ed.ac.uk/software/tracer). Each segment was run between 100 and 300 million total generations, though burnin size varied. Analysis of the PB1 segment (the largest dataset) is not presented because it failed to converge.

Accounting for the 27-year shift in sampling dates in the re-emergent H1N1 clade resulted in significantly lower variance (i.e., 95% highest posterior density width) in the tMRCA estimates, compared with the “uncorrected” analysis. The shifted dates reduced the variance in the tMRCA estimate for the re-emergent H1N1 viruses by 39% (Wilcoxon signed-rank test, *p* = 0.028) (Table 1). Moreover, including the unadjusted sampling dates also significantly increased the variance in divergence time estimation across all nodes in the phylogeny by an average of 5% (Wilcoxon signed-rank test, *p* = 0.028). Thus, even distantly related nodes were affected by the inclusion of the re-emergent H1N1 viral isolates. However, this pattern of increased variance in divergence time estimation was not seen in analysis of the M segment. The reason for this is not clear as the M segment alignment and BMCMC analysis was not remarkably different from those of the other genome segments. Statistical analyses were performed using Stata v11.0 (StataCorp LP).

**Table 1:** Highest posterior density (HPD) width in years of the node leading to the re-emergent H1N1 lineage before and after a 27-year shift in sampling dates.

Segment	HPD width (reported sampling years)	HPD width (27-year shift for re-emergent lineage)	Ratio of HPDs (reported:shift)
HA	4.48	2.55	0.57
M	5.96	6.41	1.07
NA^a^	27.31	2.26	0.08
NA-mode 1	3.17	–	0.71
NA-mode 2	4.48	–	0.49
NP	6.42	4.17	0.65
NS	5.18	3.54	0.68
PA	4.65	2.95	0.64
PB2	5.20	3.09	0.59

a NA had a bimodal distribution for the age of the node leading to re-emergent H1N1 lineage. Mode 1 was 1973–1977 and mode 2 was 1949–1953. Inclusion of either mode does not alter the pattern of significantly decreased variance following the 27-year shift.

The re-emergence of H1N1 is not the only instance in which the year of sampling does not correspond to amount of sequence evolution. Dozens of other influenza isolates have been identified as having unrealistically short branch lengths, possibly resulting from laboratory contamination, mislabeling, and/or re-introduction ([13], [14]; accidental infection of a laboratory worker: A/Canada/720/05). Many of these additional suspect sequences were also included in the analysis by Smith et al. [9]. Furthermore, other included samples are actually reassortant vaccine strains, whose segments were isolated decades apart from one another (e.g., A/New Jersey/1976 and A/Leningrad/54/1). In fact, A/Leningrad/54/1, which has an erroneous sampling date of 1954, is actually a reassortant vaccine with segments isolated in 1934 and 1977 [16], [17], [18]. This sequence alone accounts for the bimodal distribution of node ages observed in the NA analysis (Table 1), as one of the modes is not sampled after its removal. Based on the results presented here, the inclusion of these and other sequences with biologically unrealistic sampling dates can dramatically affect tMRCA estimates and should be avoided.

Our observation of increased variance when calibrating with unadjusted sampling dates prompted us to re-estimate the age of the 1977 re-emergent lineage using a dataset free of sequences with biologically unrealistic sampling dates. Therefore, additional BMCMC inference was performed on a representative sample of 99 human H1N1 viruses isolated between 1918 and 2009. The sampling age of the re-emergent isolates was adjusted by 27 years. Two independent BMCMC runs of 25 million generations were performed for each segment. Model comparison was performed via Bayes Factor in Tracer v1.5 (Text S1; Tables S1, S2, S3, S4, S5, S6, S7, and S8); differences in tMRCA estimates among models were trivial. Sequence alignments are available upon request.

**Figure 3:**
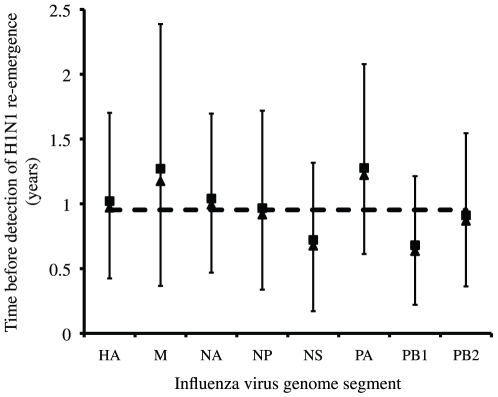
Posterior distributions for the tMRCA of the re-emergent H1N1 lineage for all eight influenza virus segments. Squares are mean values, triangles are median values, bars are 95% highest probability densities, and the dashed line is the sample size weighted average.

Smith et al. [9] placed the mean tMRCA of the re-emergent H1N1 lineage in 1974 or 1975; however, these estimates are biased by the missing 27 years of sequence evolution. According to our analysis, the re-emergent H1N1 lineage began diversifying approximately one year before it was first detected in China and Russia (sample size weighted average from [19]) (Figure 3); the posterior distributions for the tMRCA of the re-emergent lineage excludes the year of re-emergence. If the virus was circulating for up to a year before detection, then it seems difficult to assign the geographic source of re-introduction (i.e., China or Russia) based solely on surveillance in 1977. This interpretation must be treated with caution as our inference was powered to detect differences on the order of calendar years, because the date of viral isolation was measured in years and not in months or days.

We acknowledge that simply adjusting the re-emergent sampling dates by 27 years may not be an ideal method to estimate the date of re-emergence; however, the results presented here demonstrate that some correction to the biased sampling dates is needed before inferring divergence times. A new method is needed to account for samples with unrealistic sampling dates. In the case of a re-emergent clade, the amount of missing evolution along the branch leading to re-emergence could be estimated as a model parameter in a Bayesian framework. For single isolates with unrealistic sampling dates (e.g., laboratory contaminants and vaccine strains), the posterior distribution of the sampling date could be estimated during the analysis instead of being treated as a fixed value.

Re-emergence and laboratory contamination is a problem not limited to influenza virus. A similar pattern of missing decades of sequence evolution was recently observed in rabbit hemorrhagic disease virus [20]. Furthermore, using strains that have undergone long-term passage and selection in the laboratory, which is not uncommon in studies estimating viral tMRCAs, would have the opposite effect of lengthening branches [21]. It is likely that calibrating a molecular clock using these laboratory-passaged strains would also have detrimental effects on estimating tMRCAs. To ensure reliable divergence time estimates, we must start with high quality datasets.

## Supporting Information

Text S1 Model selection methods
(0.03 MB DOC)

Table S1 Bayes factor model test on HA segment.
(0.03 MB DOC)

Table S2 Bayes factor model test on M segment.
(0.03 MB DOC)

Table S3 Bayes factor model test on NA segment.
(0.03 MB DOC)

Table S4 Bayes factor model test on NP segment.
(0.03 MB DOC)

Table S5 Bayes factor model test on NS segment.
(0.03 MB DOC)

Table S6 Bayes factor model test on PA segment.
(0.03 MB DOC)

Table S7 Bayes factor model test on PB1 segment.
(0.03 MB DOC)

Table S8 Bayes factor model test on PB2 segment.
(0.03 MB DOC)
