# Supplementary material for: The Re-Emergence of H1N1 Influenza Virus in 1977: A Cautionary Tale for Estimating Divergence Times Using Biologically Unrealistic Sampling Dates
Source: PLoS One. 2010 Jun 17;5(6):e11184. doi: 10.1371/journal.pone.0011184 (PMC2887442; doi:10.1371/journal.pone.0011184)
Supplement: Text S1 — Model selection methods (0.03 MB DOC) [file pone.0011184.s001.doc]

Model selection was performed using Bayes factor, which compared the marginal likelihoods for each run. For each influenza gene segment, 3 clock models (UCED, uncorrelated exponential distribution relaxed molecular clock; UCLD, uncorrelated lognormal distribution relaxed molecular clock; strict, strict molecular clock) and 3 coalescent demographic models (Constant, constant population size; Exponential, exponential population size; BSP, Bayesian skyline plot) were compared. For gene segments that contained a single non-overlapping open-reading frame, 2 substitution models were compared (GTR+Γ_4_; SRD06, Shapiro B, Rambaut A, Drummond AJ. 2006. Choosing appropriate substitution models for the phylogenetic analysis of protein-coding sequences. Mol Biol Evol. 23:7–9.).

The UCED clock model was preferred in every case. There were no significant differences in marginal likelihoods among the coalescent demographic models. Therefore, BSP was chosen because it places the fewest constraints on the analyses. SRD06 was preferred over GTR+Γ_4_, in the segments in which it was possible to use this model.
