# Supplementary material for: The Re-Emergence of H1N1 Influenza Virus in 1977: A Cautionary Tale for Estimating Divergence Times Using Biologically Unrealistic Sampling Dates
Source: PLoS One. 2010 Jun 17;5(6):e11184. doi: 10.1371/journal.pone.0011184 (PMC2887442; doi:10.1371/journal.pone.0011184)
Supplement: Table S1 — Bayes factor model test on HA segment. (0.03 MB DOC) [file pone.0011184.s002.doc]

| **Model** | **ln P**  **(model | data)** | **SE** | **GTR+Γ_4_**  **UCED**  **BSP** | **SRD06**  **Strict**  **BSP** | **SRD06**  **UCED**  **Constant** | **SRD06**  **UCED**  **Exponential** | **SRD06**  **UCED**  **BSP** | **SRD06**  **UCLD**  **BSP** |
| --- | --- | --- | --- | --- | --- | --- | --- | --- |
| GTR+Γ_4_  UCED  BSP | -9638.1 | 0.375 | - | -31.943 | -56.506 | -57.006 | -56.974 | -52.54 |
| SRD06  Strict  BSP | -9564.549 | 0.342 | 31.943 | - | -24.563 | -25.063 | -25.031 | -20.597 |
| SRD06  UCED  Constant | -9507.991 | 0.422 | 56.506 | 24.563 | - | -0.5 | -0.468 | 3.965 |
| SRD06  UCED  Exponential | -9506.839 | 0.353 | 57.006 | 25.063 | 0.5 | - | 0.032 | 4.466 |
| SRD06  UCED  BSP | -9506.913 | 0.338 | 56.974 | 25.031 | 0.468 | -0.032 | - | 4.434 |
| SRD06  UCLD  BSP | -9517.122 | 0.474 | 52.54 | 20.597 | -3.965 | -4.466 | -4.434 | - |
