# Supplementary material for: The Re-Emergence of H1N1 Influenza Virus in 1977: A Cautionary Tale for Estimating Divergence Times Using Biologically Unrealistic Sampling Dates
Source: PLoS One. 2010 Jun 17;5(6):e11184. doi: 10.1371/journal.pone.0011184 (PMC2887442; doi:10.1371/journal.pone.0011184)
Supplement: Table S2 — Bayes factor model test on M segment. (0.03 MB DOC) [file pone.0011184.s003.doc]

| **Model** | **ln P (model | data)** | **SE** | **GTR+Γ_4_**  **Strict**  **BSP** | **GTR+Γ_4_**  **UCED**  **Constant** | **GTR+Γ_4_**  **UCED**  **Exponential** | **GTR+Γ_4_**  **UCED**  **BSP** | **GTR+Γ_4_**  **UCLD**  **BSP** |
| --- | --- | --- | --- | --- | --- | --- | --- |
| GTR+Γ_4_  Strict  BSP | -3799.552 | 0.351 | - | -15.365 | -15.577 | -15.467 | -10.061 |
| GTR+Γ_4_  UCED  Constant | -3764.172 | 0.422 | 15.365 | - | -0.211 | -0.101 | 5.304 |
| GTR+Γ_4_  UCED  Exponential | -3763.685 | 0.362 | 15.577 | 0.211 | - | 0.11 | 5.516 |
| GTR+Γ_4_  UCED  BSP | -3763.939 | 0.318 | 15.467 | 0.101 | -0.11 | - | 5.406 |
| GTR+Γ_4_  UCLD  BSP | -3776.386 | 0.362 | 10.061 | -5.304 | -5.516 | -5.406 | - |
