# Supplementary material for: The Re-Emergence of H1N1 Influenza Virus in 1977: A Cautionary Tale for Estimating Divergence Times Using Biologically Unrealistic Sampling Dates
Source: PLoS One. 2010 Jun 17;5(6):e11184. doi: 10.1371/journal.pone.0011184 (PMC2887442; doi:10.1371/journal.pone.0011184)
Supplement: Table S3 — Bayes factor model test on NA segment. (0.03 MB DOC) [file pone.0011184.s004.doc]

| **Model** | **ln P**  **(model | data)** | **SE** | **GTR+Γ_4_**  **UCED**  **BSP** | **SRD06**  **Strict**  **BSP** | **SRD06**  **UCED**  **Constant** | **SRD06**  **UCED**  **Exponential** | **SRD06**  **UCED**  **BSP** | **SRD06**  **UCLD**  **BSP** |
| --- | --- | --- | --- | --- | --- | --- | --- | --- |
| GTR+Γ_4_  UCED  BSP | -7596.283 | 0.384 | - | -40.258 | -65.733 | -66.371 | -66.111 | -61.628 |
| SRD06  Strict  BSP | -7503.586 | 0.327 | 40.258 | - | -25.475 | -26.114 | -25.853 | -21.37 |
| SRD06  UCED  Constant | -7444.928 | 0.481 | 65.733 | 25.475 | - | -0.638 | -0.378 | 4.105 |
| SRD06  UCED  Exponential | -7443.458 | 0.369 | 66.371 | 26.114 | 0.638 | - | 0.261 | 4.744 |
| SRD06  UCED  BSP | -7444.058 | 0.376 | 66.111 | 25.853 | 0.378 | -0.261 | - | 4.483 |
| SRD06  UCLD  BSP | -7454.38 | 0.397 | 61.628 | 21.37 | -4.105 | -4.744 | -4.483 | - |
