# Supplementary material for: The Re-Emergence of H1N1 Influenza Virus in 1977: A Cautionary Tale for Estimating Divergence Times Using Biologically Unrealistic Sampling Dates
Source: PLoS One. 2010 Jun 17;5(6):e11184. doi: 10.1371/journal.pone.0011184 (PMC2887442; doi:10.1371/journal.pone.0011184)
Supplement: Table S4 — Bayes factor model test on NP segment. (0.03 MB DOC) [file pone.0011184.s005.doc]

| **Model** | **ln P**  **(model | data)** | **SE** | **GTR+Γ_4_**  **UCED**  **BSP** | **SRD06**  **Strict**  **BSP** | **SRD06**  **UCED**  **Constant** | **SRD06**  **UCED**  **Exponential** | **SRD06**  **UCED**  **BSP** | **SRD06**  **UCLD**  **BSP** |
| --- | --- | --- | --- | --- | --- | --- | --- | --- |
| GTR+Γ_4_  UCED  BSP | -6701.974 | 0.409 | - | -61.787 | -77.341 | -77.457 | -77.617 | -69.707 |
| SRD06  Strict  BSP | -6559.704 | 0.343 | 61.787 | - | -15.553 | -15.67 | -15.83 | -7.919 |
| SRD06  UCED  Constant | -6523.891 | 0.396 | 77.341 | 15.553 | - | -0.117 | -0.276 | 7.634 |
| SRD06  UCED  Exponential | -6523.623 | 0.41 | 77.457 | 15.67 | 0.117 | - | -0.16 | 7.75 |
| SRD06  UCED  BSP | -6523.255 | 0.405 | 77.617 | 15.83 | 0.276 | 0.16 | - | 7.91 |
| SRD06  UCLD  BSP | -6541.469 | 0.425 | 69.707 | 7.919 | -7.634 | -7.75 | -7.91 | - |
