# Supplementary material for: The Re-Emergence of H1N1 Influenza Virus in 1977: A Cautionary Tale for Estimating Divergence Times Using Biologically Unrealistic Sampling Dates
Source: PLoS One. 2010 Jun 17;5(6):e11184. doi: 10.1371/journal.pone.0011184 (PMC2887442; doi:10.1371/journal.pone.0011184)
Supplement: Table S5 — Bayes factor model test on NS segment. (0.03 MB DOC) [file pone.0011184.s006.doc]

| **Model** | **ln P (model | data)** | **SE** | **GTR+Γ_4_**  **Strict**  **BSP** | **GTR+Γ_4_**  **UCED**  **Constant** | **GTR+Γ_4_**  **UCED**  **Exponential** | **GTR+Γ_4_**  **UCED**  **BSP** | **GTR+Γ_4_**  **UCLD**  **BSP** |
| --- | --- | --- | --- | --- | --- | --- | --- |
| GTR+Γ_4_  Strict  BSP | -3728.967 | 0.275 | - | -13.06 | -13.876 | -14.442 | -7.693 |
| GTR+Γ_4_  UCED  Constant | -3698.895 | 0.444 | 13.06 | - | -0.816 | -1.382 | 5.367 |
| GTR+Γ_4_  UCED  Exponential | -3697.017 | 0.295 | 13.876 | 0.816 | - | -0.566 | 6.183 |
| GTR+Γ_4_  UCED  BSP | -3695.714 | 0.428 | 14.442 | 1.382 | 0.566 | - | 6.749 |
| GTR+Γ_4_  UCLD  BSP | -3711.253 | 0.494 | 7.693 | -5.367 | -6.183 | -6.749 | - |
