# Supplementary material for: The Re-Emergence of H1N1 Influenza Virus in 1977: A Cautionary Tale for Estimating Divergence Times Using Biologically Unrealistic Sampling Dates
Source: PLoS One. 2010 Jun 17;5(6):e11184. doi: 10.1371/journal.pone.0011184 (PMC2887442; doi:10.1371/journal.pone.0011184)
Supplement: Table S6 — Bayes factor model test on PA segment. (0.03 MB DOC) [file pone.0011184.s007.doc]

| **Model** | **ln P**  **(model | data)** | **SE** | **GTR+Γ_4_**  **UCED**  **BSP** | **SRD06**  **Strict**  **BSP** | **SRD06**  **UCED**  **Constant** | **SRD06**  **UCED**  **Exponential** | **SRD06**  **UCED**  **BSP** | **SRD06**  **UCLD**  **BSP** |
| --- | --- | --- | --- | --- | --- | --- | --- | --- |
| GTR+Γ_4_  UCED  BSP | -8894.197 | 0.435 | - | -80.503 | -102.985 | -103.47 | -103.025 | -96.272 |
| SRD06  Strict  BSP | -8708.833 | 0.343 | 80.503 | - | -22.482 | -22.968 | -22.522 | -15.769 |
| SRD06  UCED  Constant | -8657.065 | 0.346 | 102.985 | 22.482 | - | -0.485 | -0.04 | 6.713 |
| SRD06  UCED  Exponential | -8655.948 | 0.367 | 103.47 | 22.968 | 0.485 | - | 0.445 | 7.198 |
| SRD06  UCED  BSP | -8656.973 | 0.382 | 103.025 | 22.522 | 0.04 | -0.445 | - | 6.753 |
| SRD06  UCLD  BSP | -8672.523 | 0.393 | 96.272 | 15.769 | -6.713 | -7.198 | -6.753 | - |
