# Supplementary material for: The Re-Emergence of H1N1 Influenza Virus in 1977: A Cautionary Tale for Estimating Divergence Times Using Biologically Unrealistic Sampling Dates
Source: PLoS One. 2010 Jun 17;5(6):e11184. doi: 10.1371/journal.pone.0011184 (PMC2887442; doi:10.1371/journal.pone.0011184)
Supplement: Table S7 — Bayes factor model test on PB1 segment. (0.03 MB DOC) [file pone.0011184.s008.doc]

| **Model** | **ln P (model | data)** | **SE** | **GTR+Γ_4_**  **Strict**  **BSP** | **GTR+Γ_4_**  **UCED**  **Constant** | **GTR+Γ_4_**  **UCED**  **Exponential** | **GTR+Γ_4_**  **UCED**  **BSP** | **GTR+Γ_4_**  **UCLD**  **BSP** |
| --- | --- | --- | --- | --- | --- | --- | --- |
| GTR+Γ_4_  Strict  BSP | -9841.805 | 0.347 | - | -32.209 | -32.28 | -32.739 | -25.785 |
| GTR+Γ_4_  UCED  Constant | -9767.64 | 0.395 | 32.209 | - | -0.07 | -0.529 | 6.424 |
| GTR+Γ_4_  UCED  Exponential | -9767.478 | 0.421 | 32.28 | 0.07 | - | -0.459 | 6.495 |
| GTR+Γ_4_  UCED  BSP | -9766.422 | 0.339 | 32.739 | 0.529 | 0.459 | - | 6.954 |
| GTR+Γ_4_  UCLD  BSP | -9782.433 | 0.417 | 25.785 | -6.424 | -6.495 | -6.954 | - |
