# Supplementary material for: The Re-Emergence of H1N1 Influenza Virus in 1977: A Cautionary Tale for Estimating Divergence Times Using Biologically Unrealistic Sampling Dates
Source: PLoS One. 2010 Jun 17;5(6):e11184. doi: 10.1371/journal.pone.0011184 (PMC2887442; doi:10.1371/journal.pone.0011184)
Supplement: Table S8 — Bayes factor model test on PB2 segment. (0.03 MB DOC) [file pone.0011184.s009.doc]

| **Model** | **ln P**  **(model | data)** | **SE** | **GTR+Γ_4_**  **UCED**  **BSP** | **SRD06**  **Strict**  **BSP** | **SRD06**  **UCED**  **Constant** | **SRD06**  **UCED**  **Exponential** | **SRD06**  **UCED**  **BSP** | **SRD06**  **UCLD**  **BSP** |
| --- | --- | --- | --- | --- | --- | --- | --- | --- |
| GTR+Γ_4_  UCED  BSP | -10317.636 | 0.441 | - | -95.647 | -125.237 | -125.773 | -125.333 | -119.96 |
| SRD06  Strict  BSP | -10097.402 | 0.284 | 95.647 | - | -29.59 | -30.127 | -29.686 | -24.313 |
| SRD06  UCED  Constant | -10029.267 | 0.393 | 125.237 | 29.59 | - | -0.536 | -0.096 | 5.277 |
| SRD06  UCED  Exponential | -10028.032 | 0.416 | 125.773 | 30.127 | 0.536 | - | 0.441 | 5.813 |
| SRD06  UCED  BSP | -10029.047 | 0.409 | 125.333 | 29.686 | 0.096 | -0.441 | - | 5.373 |
| SRD06  UCLD  BSP | -10041.418 | 0.4 | 119.96 | 24.313 | -5.277 | -5.813 | -5.373 | - |
